# Increased Risk of Herpes Zoster in Rheumatoid Arthritis Not Only Due to JAK Inhibitors—Study of 392 Patients from Single University Center

**DOI:** 10.3390/jcm13113121

**Published:** 2024-05-26

**Authors:** Lucía C. Domínguez-Casas, Carmen Lasa-Teja, Iván Ferraz-Amaro, Santos Castañeda, Ricardo Blanco

**Affiliations:** 1 Rheumatology, Hospital Universitario Marqués de Valdecilla, 39008 Santander, Spain; carmenlasa19@gmail.com; 2 Rheumatology, Hospital Universitario de Canarias, 38320 Tenerife, Spain; iferrazamaro@hotmail.com; 3 Rheumatology, Hospital Universitario de La Princesa, IIS-Princesa, 28006 Madrid, Spain; scastas@gmail.com

**Keywords:** rheumatoid arthritis, herpes zoster, infection, biological therapy, JAK inhibitors

## Abstract

**Background/Objectives**: Patients with rheumatoid arthritis (RA) have an increased risk of infection. Their risk of presenting herpes zoster (HZ) is 1.5–2 times higher than immunocompetent individuals and disseminated presentation is more frequent. Our aim was to analyze the prevalence and general features of HZ in RA patients. **Methods**: This was a prospective study of 392 RA patients included in the vaccination program of our hospital between 2011 and 2016, and follow-up continued until December 2020. A diagnosis of HZ was made according to clinical manifestations: skin rash, blisters, paresthesia, and local pain in one or more dermatomes. **Results**: We studied 392 participants (309 women/83 men), mean age 59 ± 13 years. Every patient was followed-up over a mean period of 137 ± 110 months (range: 42 months-42 years). HZ infection was observed in 30 of 392 (25 women/5 men) patients, age (mean ± SD) 64.7 ± 11.8 years. Prevalence was 7.65% in this period and the incidence rate was 13.22/1000 patients/year. Three patients had facial involvement, one had optic involvement, and one patient presented disseminated HZ. Seven patients presented post herpetic neuralgia treated with gabapentinoids. The main features of RA of these 30 patients were: positive RF (n = 17; 56.6%), positive anti-CCP (n = 13; 43.3%), and erosive disease (n = 10; 33.3%). At HZ infection, the treatments were glucocorticoids (n = 19; 63.3%), conventional DMARDs (n = 15; 50%), biological DMARDs (n = 15; 50%), tofacitinib (n = 2; 6.6%), and upadacitinib (n = 1; 3.3%). **Conclusions**: HZ is a relatively frequent viral complication in RA patients. In our series, one patient presented disseminated HZ and nearly 25% of patients had post-herpetic neuralgia. Including a HZ vaccine in our vaccination program for RA patients may be beneficial.

## 1. Introduction

Rheumatoid arthritis (RA) carries an increased risk of infection [1,2]. This increased risk of infection is associated with the immune dysregulation due to the disease itself and the immunomodulation drugs used to treat the disease. Furthermore, there are other risk factors such as age, comorbidity, and concomitant treatments [3,4,5,6].

Varicella zoster virus (VZV) may produce two different diseases. The primary infection that usually appears in childhood is varicella or chickenpox, and it is characterized by a skin rash and small pruriginous blisters on the chest, back, and face, which subsequently spread to other regions [7,8,9]. Second, there is herpes zoster (HZ) or shingles, which is caused by the reactivation of latent infection from the virus. In fact, anyone that has suffered from varicella can develop HZ [10]. After the first infection, viral particles of VZV settle in the cranial and dorsal root ganglia and cell-mediated immunity is created by specific antibodies and CD4+ T cells [11].

HZ presents with dysesthesia in the sensory region for the affected ganglia, followed by a skin rash and blisters. In 2–3 days, these blisters ulcerate and form scabs that may last for months. After the acute phase, neuropathic pain may remain for weeks and months, causing post-herpetic neuralgia (PHN) in 20–30% of patients, which represents an important morbidity requiring pharmacological treatment in most cases [12,13].

The diagnosis of HZ is usually clinical, but can be confirmed by the demonstration of VZV-DNA by PCR in a sample of the blisters’ contents [14]. HZ infections have been associated with many triggers. Between them, we find intercurrent acute illnesses, malignancy, emotional stress, and the use of some types of medication such as immunosuppressive agents, particularly glucocorticoids (GC) and methotrexate (MTX) [15,16,17,18].

In general population, the incidence of VZV reactivation is 3–5/1000 patients-year, with a similar incidence in the US, Europe, and Asia [19]. This risk is increased in aged people (6–8/1000/year in people over 60 years and 8–12/1000/years in individuals older than 80 years), mainly due to the decline in cell-mediated immunity related to age [20,21].

After 85 years, the risk of developing HZ is near 50% and sequelae are much more common. The same occurs in immunocompromised patients, such as those with bone narrow or solid organ transplantation, lymphoproliferative diseases, leukemia, and human immunodeficiency virus (HIV) infection. In the same group of risk are patients with rheumatic or systemic autoimmune diseases [19,22]. In fact, RA patients have a 1.5 to 2 times higher risk of presenting HZ than immunocompetent individuals. Furthermore, disseminated forms and post-herpetic sequelae are much more likely in these patients [23].

Taking into account all these considerations, the aims of this study were to assess the incidence and clinical characteristics of HZ in a group of RA patients followed in a single university hospital, to establish the risk factors for developing this virus reactivation, and then to establish the need to include the HZ vaccine in the vaccination programs of our health system.

## 2. Materials and Methods

### 2.1. Design and Enrollment Criteria

We performed a prospective study of patients diagnosed with RA from a single hospital (Marqués de Valdecilla University Hospital, Santander, Spain) who were consecutively included in the vaccination program of the hospital over a 5-year period (from October 2011 to October 2016). These patients were later followed-up until December 2020. RA was diagnosed according to the EULAR/ACR 2010 criteria [24]. Patients diagnosed before 2010 were reevaluated and the diagnosis was confirmed with these criteria.

HZ was diagnosed according to cutaneous manifestations of skin rash and blisters, and the occurrence of neuropathic pain in one (localized) or several (disseminated) dermatomes was recorded. The presence of cutaneous manifestations was evaluated with an exhaustive physical exploration performed by a rheumatologist or a general physician. In all doubtful cases, patients were also assessed by an experienced dermatologist. Sequelae were also checked out in all these patients [14,25].

### 2.2. Outcome Variables

Outcome variables were recorded at baseline and at the moment of HZ infection. General variables included age, sex, time of evolution of RA, and administered therapies.

The main outcome variables of RA were related to the severity of the disease. Clinical manifestations were the presence of erosions and extra-articular manifestations such as rheumatoid nodules, associated interstitial lung disease, Sjögren’s syndrome, and rheumatoid vasculitis. Laboratory findings included positivity for rheumatoid factor (RF) and anti-citrullinated cyclic peptide antibodies (anti-CCP).

Treatment with prednisone, conventional synthetic (cs) disease-modifying antirheumatic drugs (DMARDs), biological (b) DMARDs, and JAK inhibitors (JAKinh) prior to and at the moment of the shingles occurrence was recorded and later analyzed.

The associated comorbidities in these patients were also collected and analyzed to find risk factors. These comorbidities included hypertension, hypercholesterolemia, diabetes mellitus, and active smoking. Many triggers have been reported in patients with HZ. The most commonly referred to are emotional stress, the use of medications (immunosuppressive drugs), intercurrent acute or chronic illnesses, and the presence of malignancy [15]. All of them were searched in our patients.

The localization of HZ was described by the rheumatologist, general physician, or dermatologist in every case. Adequate responses to antiviral treatment were evaluated in the two weeks following the diagnosis of HZ. The presence of sequelae, such as PHN or visual alterations in the cases of facial involvement, and the need for neuropathic pain killers were recorded in this period and followed-up until symptoms disappeared.

We also calculated the prevalence and incidence rate of HZ in our cohort.

### 2.3. Data Collection

The data of the patients were collected from the clinical records of our hospital and from the informatics programs of General Practitioners. These data included clinical and laboratory data, diagnosis, and the pharmacological agents used in the management of RA. Manifestations of HZ, localization, the antiviral treatment used, and sequelae were also registered. The data were reviewed to confirm the diagnosis and then stored in a computerized data file.

### 2.4. Statistical Analysis

The results are expressed as mean ± standard deviation (SD) for variables with a normal distribution, or as median and interquartile range [IQR] [25th, 75th] for those not normally distributed. To compare continuous variables, the Wilcoxon signed-rank test was used. For the analysis of dichotomous variables, the chi-square test or the Fisher exact test were used. To check for risk factors of HZ, a Cox regression analysis was performed. Statistical analysis was performed using the Stata software, version 17/SE (StataCorp, College Station, TX, USA). A *p*-value of < 0.05 was considered as statistically significant.

## 3. Results

We studied 392 RA patients that were included consecutively in the vaccination program of the Preventive Medicine department of our hospital from October 2011 to October 2016. The mean follow-up was 11 years. None of the included patients had previously received the vaccination for HZ.

### 3.1. Demographic and Clinical Features of the Participants

The demographic and disease-related characteristics of the participants are shown in Table 1. The mean age was 59 ± 13 years and 79% of patients were female. Current smoking, hypertension, dyslipidemia, and diabetes mellitus were present in 40%, 42%, 38%, and 14% of patients, respectively. Positivity for RF and anti-CCP was 57% and 53%, respectively. In total, 145 patients (37%) presented an erosive disease. The most important extra-articular manifestations were subcutaneous nodules (6%), interstitial lung disease (5%), Sjögren’s syndrome (5%), and rheumatoid vasculitis (6%).

A total of 30 patients (25 women/5 men) developed HZ during follow-up. The mean age of the patients at the moment of HZ was 64.7 ± 11.8 years. The mean follow-up duration was 45 months, with a minimum period of 4 months and a maximum of 7 years. The main demographics, clinical data, and treatments of these 30 patients at the moment of HZ infection are shown in Table 2.

In the group with HZ, most of the patients presented important comorbidities such as a high blood pressure (60%), dyslipidemia (47%), and diabetes mellitus (20%). Eleven patients were active smokers. 

Half of the HZ patients were found to be positive for RF (57%) or anti-CCP (43%), and almost half had an erosive RA (33%). In regard to extra-articular manifestations, three patients presented interstitial lung disease (10%), one patient associated with Sjögren´s syndrome (3%), and two patients with rheumatoid vasculitis (7%). One patient presented rheumatoid nodules (3%).

Comparisons between the main clinical characteristics and RA treatments of both groups, non-HZ and HZ patients, at baseline visit, are shown in Table 1.

There were no differences in either age, time of evolution of RA, or sex, nor in active smoking, dyslipidemia, and diabetes mellitus. Hypertension was statistically more common in patients with HZ in our RA cohort (*p* = 0.039). RA-related features (RF, anti-CCP, the presence of erosions, associated lung disease, Sjögren´s syndrome, rheumatoid nodules, and rheumatoid vasculitis) did not show differences at baseline.

### 3.2. Therapies Administered at Baseline

A comparison of RA treatments was also made. More than half of the patients of our cohort were receiving treatment with GC at baseline with a mean prednisone-equivalent dose of 3.75 (0–7.5) mg/day in the HZ group versus 5 (0–5) in the other group, with no significant differences. Conventional synthetic DMARDs were the drugs more frequently used in both groups, preferably MTX, leflunomide, sulfasalazine, and azathioprine, with no statistical differences between both groups. Likewise, there were no differences in baseline treatment with bDMARDs in both groups. In contrast, the number of TNF inhibitors used in the HZ group was higher than that in the non-HZ group (*p* = 0.014), especially with etanercept (*p* = 0.033). Regarding the duration (months or years) of exposure to biological or targeted therapies, no significant differences were observed between the patients who suffered from HZ and those who did not.

### 3.3. Herpes Zoster Infection Characteristics

**Figure 1:**
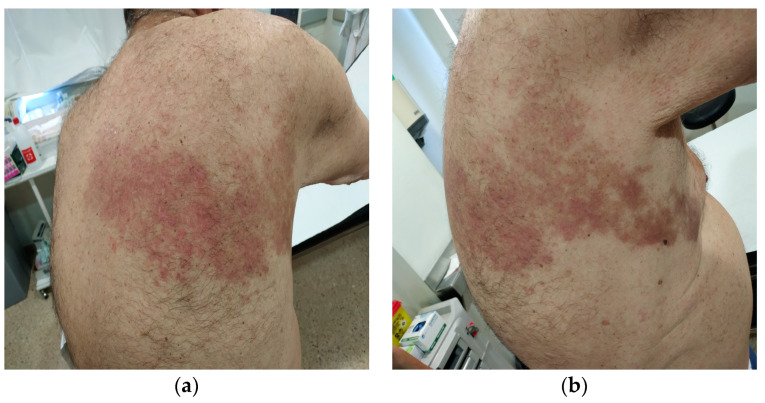
Sequelae of dorsal herpes zoster in a patient with RA. (**a**) Posterior view. (**b**) Lateral view.

The HZ infection characteristics are collected in Table 2 and Supplementary Table S1. The main locations for HZ were in the trunk [intercostal (n = 6), dorsal (n = 4), lumbar (n = 3), abdominal (n = 3), intermammary fold (n = 1), and submmamaryfold (n = 1)], followed by the head, neck [cervical (n = 1), ophthalmic (n = 1), facial (n = 1), and trigeminal (n = 1)], and extremities [left upper extremity (n = 1) and gluteus (n = 1)]. One patient presented a disseminated form of HZ involving three different zones of the body. We did not identify the location of HZ in five patients. PHN, as the principal sequelae, was found in seven patients. Neuropathic pain killers such as gabapentinoids (pregabalin and gabapentin) were needed for several months in these patients. One of them also presented cutaneous sequelae, skin erythematosus, and painful and pruriginous lesions, as we can see in Figure 1.

The patient with ophthalmic involvement suffered from visual loss for a week with a later complete resolution after that time.

HZ was treated only with topical treatment in two patients, and five were not treated due to a late diagnosis. Two patients received both topical and systemic antiviral agents, and twenty-one patients received only systemic treatment. The main antiviral drugs used were brivudine and famciclovir, both of them in seven patients. The other treatments were aciclovir (n = 6) and valaciclovir (n = 3).

Interestingly, we did not identify any trigger for HZ development in our patients. Although no specific screening was conducted to rule out malignancies in patients with extended HZ, these patients did not present symptoms or laboratory test abnormalities, nor did they develop malignancies during the follow-up. Remarkably, no patients reported a primary infection for VZV during the follow-up period.

### 3.4. RA Treatment in HZ Infection

Concerning RA treatment, more than the half of the patients (63.3%) were taking GC at the time of VZV reactivation, and the mean dose was 7.5 (5–10) mg/day.

Almost every patient was under treatment with csDMARDs (n = 9), bDMARDs (n = 10), or JAK inhibitors either in monotherapy (n = 2) or in combination (n = 7). One patient was receiving only GC, and another was not receiving any treatment for RA at that moment.

The csDMARDs used were: MTX (n = 11), leflunomide (n = 3), sulfasalazine (n = 1), and azathioprine (n = 1). One patient was taking leflunomide and MTX in combination.

Eight patients were receiving TNF-α antagonists, four in combination with csDMARDs. Etanercept (ETN) (n = 4) was the most common drug used, being given at the standard dose. Adalimumab (ADA) (n = 1) was used at the standard dose too. Golimumab (GLM) (n = 2) was used at the standard dose in one patient and optimized in another patient (50 mg subcutaneously every 45 days). Certolizumab pegol was used in a single patient at the standard dose.

In the remaining patients under bDMARDs, the non-TNFα drugs used were tocilizumab (n = 3), abatacept (n = 3), sarilumab (n = 1), and JAK inhibitors in three patients (tofacitinib in two cases and upadacitinib in the other one). Every one of these drugs was administrated at the standard dose.

### 3.5. Multivariate Analysis

Cox regression revealed that hypertension was the only comorbidity associated with VZV reactivation [Hazard Ratio (HR) 2.25; 95% CI: 1.09–4.68; *p* = 0.029]. Regarding RA treatment, only exposure to etanercept showed a relation with HZ infection in our patients (HR 2.04, 95% CI: 0.99–4.18; *p* = 0.05).

In Table 3, we summarize the proposed predictive factors of HZ development in our cohort of patients with RA.

## 4. Discussion

In this study, we presented 30 cases of HZ in a series of 392 patients with RA included in the vaccination program of our hospital over a 5-year period.

HZ or shingles is the result of the reactivation of VZV that rests latent in the cranial and dorsal root ganglia after a primary infection, normally in childhood [7,8,9,10,11,12,13,14,15,16,17,18,19].

HZ is a relatively frequent infection in the general population, at 3–5/1000 patients/year, and the estimated incidence in immunocompromised people, such as RA patients, is 1.5–2 times higher [1,2]. In 2006, Pappas et al. performed an observational study including 28,852 RA patients, with a HZ incidence of 7.7/1000 patients a year [17]. One year later, Smitten et al. studied the incidence of HZ in two retrospective cohorts of RA (122,272 and 38,621 patients), with incidence rates of 9.83 and 3.71/1000 patients a year, respectively [2]. Other classic cohorts are McDonald et al. (2009), including 20,357 patients with RA and other musculoskeletal diseases, with an incidence rate of 9.96/1000 patients a year [26]; Chen et al. (2011), including 11,446 patients with immune-mediated diseases at an incidence rate of 12.24/1000 patients a year; Wolfe et al. (2015) [27], including 10,614 RA patients at an incidence rate of 13.20/1000 patients a year; and Harada et al. (2017) [28], including 1987 RA patients at an incidence rate of 6.66/1000 patients a year. The incidence rate for HZ in our RA cohort was 13.2 per 1000 patients a year, a little higher than the other cohorts in the above-mentioned publications.

Remarkably, not only does the acute infection by HZ cause important morbidity, but it also causes sequelae such as PHN, which is frequent in this type of patients. Disseminated variants of HZ affecting two or more dermatomes and internal organs are also more frequent in RA patients. Severe complications such as skin rash bacterial super-infection or Ramsay-Hunt syndrome are more likely in immunocompromised patients, and loss of vision when ophthalmic involvement occurs is also possible [17]. As expected, the group of RA patients that developed HZ in our cohort was older (mean age of 65 years) and the time of evolution of the RA was longer (mean duration of 13 years).

Over the last few years, new treatments for RA and other immune-mediated inflammatory diseases have increased the risk of the reactivation of VZV as the population ages [2,4,20]. 

Several studies have demonstrated that high doses of GC are associated with an increased risk of infections, especially opportunistic infections such as the reactivation of VZV [6,17,29]. In our series, more than half of the patients with HZ (63.3%) were under treatment with GC when shingles occurred, although the mean dose was relatively low [7.5 (5–10) mg/day].

The reason for the increased risk of HZ in patients treated with anti-TNF drugs is not clear. In 2007, Smitten et al. founded a slightly elevated risk of HZ in patients treated with conventional and biological DMARDs, compared to RA patients with no therapy [2]. Two years later, one study in the US showed that the use of adalimumab or etanercept seemed to protect against the reactivation of VZV, and the risk with infliximab was significantly low [26]. Interestingly, in the RABBIT registry, there were no consistent data. The incidence of HZ was similar in the monoclonal antibodies (adalimumab and infliximab), etanercept, and non-biologic DMARD groups, but in the multivariate analysis, the risk was significantly higher in patients treated with TNF-αantagonists [30].

In our HZ cohort, eight patients were under treatment with anti-TNFα at the moment of the virus reactivation, with etanercept being the most frequently used (n = 4), followed by golimumab (n = 2), adalimumab (n = 1), and certolizumab pegol (n = 1). Furthermore, the number of anti-TNF drugs used in this group was significantly higher than in the other group (*p* = 0.014), especially etanercept (*p* = 0.033).

For other bDMARD agents, the risk of HZ has not been systematically evaluated. The results of several studies are not consistent, but the general results suggest that the use of other biological drugs may also increase the risk of shingles [31,32,33]. In fact, in our HZ cohort, three patients were taking tocilizumab, three were taking abatacept, and one was taking sarilumab at the moment of the virus reactivation.

JAK inhibitors are the latest promising treatments for RA, but the risk of HZ is also very high with them. Many reports and clinical trials have demonstrated an increased risk of VZV reactivation, especially with tofacitinib. This increased risk is due to the mechanism of action of these molecules. The inhibition of Janus kinases 1, 2, and 3 decreases the functionality of Th1 cells and blocks specific cytokines that protect from the infection or reactivation of herpes virus. Interestingly, the risk of viral infections is related to the dose in all these therapies [34,35,36,37,38,39,40].

In our group of patients with HZ, two patients were under treatment with tofacitinib and one was being treated with upadacitinib at the moment of shingles.

Currently, we know that vaccination is an essential point for preventing HZ infection. Nonetheless, until recently, vaccines to prevent the reactivation of this virus were normally non-standardized in patients with RA. In fact, in our cohort, no patient had been vaccinated for this virus at the moment of diagnosis.

Nowadays, there are two vaccines available for HZ infection. Zostavax^®^ is a live-attenuated vaccine, similar to one of the varicella vaccines administered in childhood, but fourteen times more potent in order to be able to create immunity in older or suppressed immune systems [41].

Shingrix^®^ is the last approved HZ vaccine. It is a recombinant vaccine that contains the VZV glycoprotein E and the AS01B adjuvant system. Its mechanism of action is similar to Zostavax, increasing the production of specific CD4+T-cells. Studies have demonstrated that the response persists for at least 3 years, even in immunocompromised patients [42]. The main advantage of this vaccine is the possibility of administration at any moment during disease. Recent reports suggest that the recombinant vaccine is superior to the live-attenuated vaccine in reducing the number of cases of HZ and sequelae such as PHN.

The last EULAR recommendations for vaccination (2019) state that HZ vaccinations should be considered in adult patients with autoimmune inflammatory rheumatic diseases, but these recommendations are not homogeneous in most hospitals yet [43]. In fact, in our country, this vaccine is currently approved only for patients older than 65 years or for patients under certain therapies such as JAK inhibitors.

One of the most important limitations of this study is that, due to the type of study and information collection, as well as the heterogeneity of the physicians involved throughout the study period, some important data were unevenly collected, especially those related to the personal history of the patients and whether the recruited subjects had previously passed VZV or not.

## 5. Conclusions

Overall, we can conclude that risk of HZ is higher in immunocompromised patients such as RA patients, who are more likely to suffer from complications or sequelae like PHN. In our series, despite the fact that the number of patients under treatment with JAK inhibitors was low, the incidence rate was high. Thus, we think that a new recombinant vaccine for HZ should be recommended in vaccination programs to all patients with RA.

## Figures and Tables

**Table 1:** Demographics and disease-related data differences at baseline between patients developing herpes zoster infection or not.

	RA Patients (N = 392)	RA Patients with HZ (N = 30)	RA Patients without HZ (N = 362)	*p*-Value
Age (years) (mean ± SD)	59 ± 13	61 ± 13	59 ± 13	0.36
Time of evolution of RA, (months) (mean ± SD)	136.9 ± 109.8	158 ± 118	135.3 ± 109.2	0.33
Women, n (%)	309 (79)	25 (83)	284 (78)	0.53
Smokers, n (%)	155 (40)	11 (37)	144 (40)	0.74
Hypertension, n (%)	165 (42)	18 (60)	147 (41)	**0.039**
Diabetes mellitus, n (%)	55 (14)	6 (20)	49 (14)	0.33
Dyslipidemia, n (%)	148 (38)	14 (47)	134 (37)	0.30
Positive RF, n (%)	223 (57)	17 (57)	206 (57)	0.98
Positive anti-CCP Abs, n (%)	206 (53)	13 (43)	193 (53)	0.29
Erosive disease, n (%)	145 (37)	10 (33)	135 (37)	0.67
Subcutaneous nodules, n (%)	23 (6)	1 (3)	22 (6)	0.57
Interstitial lung disease, n (%)	20 (5)	3 (10)	17 (5)	0.21
Sjögren syndrome, n (%)	20 (5)	1 (3)	19 (5)	0.65
Vasculitis, n (%)	23 (6)	2 (7)	21 (6)	0.85
Baseline use of therapies				
Prednisone				
N (%)	228 (58)	16 (55)	212 (59)	0.72
Dose, mg/day	5 (0–5)	5 (0–5)	3.75 (0–7.5)	0.92
*Conventional synthetic DMARDs, n (%)*				
Methotrexate	252 (64)	20 (67)	232 (64)	0.78
Leflunomide	38 (10)	2 (7)	36 (10)	0.56
Sulfasalazine	12 (3)	1 (3)	11 (3)	0.61
*Biological DMARDs, n (%)*				
Any anti-TNFα	92 (23)	9 (30)	83 (23)	0.38
Adalimumab	35 (10)	3 (10)	32 (9)	0.74
Etanercept	40 (10)	5 (17)	35 (10)	0.23
Infliximab	7 (2)	0 (0)	7 (2)	0.99
Golimumab	43 (11)	4 (13)	39 (11)	0.56
CertolizumabPegol	1 (0)	0 (0)	1 (0)	0.99
Tocilizumab	16 (4)	1 (3)	15 (4)	0.99
Rituximab	11 (3)	2 (7)	9 (2)	0.20
Abatacept	4 (1)	0 (0)	4 (1)	0.99
Accumulated treatment for RA				
Conventional DMARDs (not concomitant), n (%)				
Methotrexate	351 (90)	26 (87)	325 (90)	0.81
Leflunomide	126 (32)	12 (40)	114 (31)	0.34
Sulfasalazine	90 (23)	7 (23)	83 (23)	0.96
Cyclosporine A	11 (2)	0 (0)	11 (3)	0.99
Gold salts	37 (9)	3 (10)	34 (9)	0.75
Biological DMARDs, n (%)				
Any anti-TNF	260 (66)	26 (87)	234 (65)	0.014
Adalimumab	145 (37)	14 (47)	131 (36)	0.25
Etanercept	139 (35)	16 (53)	123 (34)	0.033
Infliximab	40 (10)	5 (17)	35 (10)	0.22
Golimumab	33 (8)	3 (10)	30 (8)	0.73
CertolizumabPegol	13 (3)	1 (3)	12 (3)	0.99
Tocilizumab	134 (34)	15 (50)	119 (33)	0.057
Rituximab	71 (18)	9 (30)	62 (17)	0.079
Abatacept	53 (14)	6 (20)	47 (13)	0.28
Sarilimumab	3 (1)	1 (1)	2 (1)	0.093
*JAK inhibitors, n (%)*	42 (11)	4 (13)	38 (11)	0.63
Tofacitinib	16 (4)	2 (1)	14 (4)	0.46
Baricitinib	32 (8)	2 (7)	30 (8)	0.99
Upadacitinib	4 (1)	1 (3)	3 (1)	0.27

Abbreviations (in alphabetical order): CCP: cyclic citrullinatedproteins; DMARDs: disease-modifying anti-rheumatic drugs; HZ: herpes zoster; JAK: Janus kinase; N (n): number; RA: rheumatoid arthritis; TNF: tumor necrosis factor; and SD: standard deviation.

**Table 2:** Main demographics, clinical data, and treatments of 30 patients with herpes zoster.

Case	Sex	Age at HZ	HZ Location	HZ Treatment	HZ Antiviral Treatment	Post-Herpetic Neuralgia	Concomitant RA Treatment
1	Female	71	Upper extremity (left)	Unknown	-	No	Corticosteroids/MTX/TCZ
2	Female	62	Intercostal (right)	Systemic antiviral	Brivudine	No	Corticosteroids/GLM
3	Male	70	Intercostal (right)	Systemic antiviral	Famciclovir	Yes	GLM
4	Female	84	Unknown	Topic	-	No	Corticosteroids/MTX
5	Female	68	Dorsal (right)	None	-	Yes	Corticosteroids/ABA
6	Female	78	Lumbar (left)	Systemic antiviral	Brivudine	No	Corticosteroids/MTX
7	Male	72	Intercostal (left)	None	-	No	ETN
8	Female	79	Abdominal	Systemic antiviral	Brivudine	Yes	Corticosteroids/MTX
9	Female	36	Unknown	Systemic antiviral	Aciclovir	No	Corticosteroids/MTX
10	Female	51	Disseminated	Systemic antiviral	Famciclovir	Yes	Corticosteroids
11	Female	66	Dorsal (left)	None	-	No	Corticosteroids/MTX/ADA
12	Male	82	Ophthalmic (left)	Topic	-	No	Corticosteroids/ABA
13	Female	45	Abdominal	Systemic antiviral	Brivudine	No	SARI
14	Female	52	Unknown	Topic/Systemic antiviral	Aciclovir	No	Corticosteroids/TCZ
15	Female	63	Cervical	None	-	No	MTX
16	Female	71	Submammary fold (left)	Systemic antiviral	Famciclovir	No	Corticosteroids/MTX
17	Female	68	Intercostal (left)	Systemic antiviral	Brivudine	Yes	None
18	Female	53	Intermammary fold	Systemic antiviral	Brivudine	No	TOFA
19	Female	75	Lumbar	Systemic antiviral	Valaciclovir	No	Corticosteroids/MTX/UPA
20	Female	58	Unknown	Topic/Systemic antiviral	Famciclovir	No	Corticosteroids
21	Male	73	Intercostal (left)	Systemic antiviral	Aciclovir	Yes	Corticosteroids/ABA
22	Female	40	Dorsal (left)	Systemic antiviral	Famciclovir	No	Corticosteroids/AZA/CZP
23	Female	58	Abdominal	Systemic antiviral	Famciclovir	No	Corticosteroids/TCZ
24	Female	73	Lumbar (right)	Systemic antiviral	Aciclovir	No	Corticosteroids/LFN
25	Female	66	Unknown	Systemic antiviral	Aciclovir	Yes	TOFA
26	Male	57	Intercostal (left)	Systemic antiviral	Brivudine	No	Corticosteroids/SSZ/ETN
27	Female	61	Dorsal (left)	Systemic antiviral	Aciclovir	No	LFN/ETN
28	Female	63	Facial (right)	Systemic antiviral	Famciclovir	No	MTX
29	Female	67	Gluteus (left)	Systemic antiviral	Valaciclovir	No	Corticosteroids/MTX/LFN
30	Female	79	Trigeminal (left)	Systemic antiviral	Valaciclovir	No	ETN

Abbreviations (in alphabetical order): ABA: abatacept; ADA: adalimumab; AZA: azathioprine; CZP: certolizumabpegol; ETN: etanercept; GLM: golimumab; HZ: herpes zoster; LFN: leflunomide; MTX: methotrexate; RA: rheumatoid arthritis; SARI: sarilumab; SSZ: sulfasalazine; TCZ: tocilizumab; TOFA: tofacitinib; and UPA: upadacitinib.

**Table 3:** Predictive factors of herpes zoster development in our cohort of patients with rheumatoid arthritis.

	HR (95% CI)	*p*
Age (years) (mean ± SD)	1.02 (0.99–1.05)	0.21
Time of evolution of RA (months) (mean ± SD)	1.002 (0.99–1.005)	0.31
Women, n (%)	1.31 (0.50–3.42)	0.58
Active smokers, n (%)	0.90 (0.43–1.90)	0.79
Hypertension, n (%)	2.25 (1.09–4.68)	0.029
Diabetes mellitus, n (%)	1.70 (0.70–4.17)	0.24
Dyslipidemia, n (%)	1.50 (0.73–3.08)	0.27
Positive RF, n (%)	0.96 (0.46–1.97)	0.91
Positive anti-CCP, n (%)	0.67 (0.33–1.38)	0.28
Erosions, n (%)	0.86 (0.40–1.85)	0.71
Subcutaneous nodules, n (%)	0.53 (0.07–3.92)	0.54
Interstitial lung disease, n (%)	2.10 (0.64–6.94)	0.22
Sjögren syndrome, n (%)	0.62 (0.08–4.52)	0.63
Number of conventional synthetic DMARDs (not concomitant)		
Methotrexate	0.77 (0.29–2.06)	0.60
Leflunomide	1.33 (0.67–2.66)	0.42
Sulfasalazine	1.08 (0.46–2.51)	0.87
Gold salts	1.04 (0.32.3.44)	0.95
Biological DMARDs, n (%)		
Any anti-TNF	1.12 (0.51–2.47)	0.78
Adalimumab	1.21 (0.59–2.50)	0.60
Etanercept	2.04 (0.99–4.18)	0.05
Infliximab	1.88 (0.72–4.90)	0.20
Golimumab	1.05 (0.32–3.48)	0.93
Certolizumab Pegol	0.98 (0.13–7.18)	0.98
Tocilizumab	1.55 (0.75–3.17)	0.23
Rituximab	1.94 (0.88–4.22)	0.99
Abatacept	1.46 (0.59–3.57)	0.41
Sarilumab	3.90 (0.53–28.62)	0.18
JAK inhibitors	1.46 (0.56–3.82)	0.44
Tofacitinib	1.34 (0.32–5.64)	0.69
Baricitinib	0.73 (0.17–3.07)	0.67
Upadacitinib	4.14 (0.56–30.59)	0.16

Abbreviations (in alphabetical order): anti-CCP: anti-cyclic citrullinated protein antibodies; CI: confidence interval; DMARDs: disease-modifying anti-rheumatic drugs; HR: hazard ratio; JAK: Janus kinase; N (n): number; RF: rheumatoid factor; TNF: tumor necrosis factor; and SD: standard deviation.

## Data Availability

Data is unavailable due to privacy.

## Supplementary Materials

The following supporting information can be downloaded at: https://www.mdpi.com/article/10.3390/jcm13113121/s1. Supplementary Table S1. Herpes zoster infection characteristics and therapy for rheumatoid arthritis when infection appeared.

## References

[1] Winthrop K.L., Tanaka Y., Lee E.B., Wollenhaupt J., Al Enizi A., Azevedo V.F., Curtis J.R. (2022). Prevention and management of herpes zoster in patients with rheumatoid arthritis and psoriatic arthritis: A clinical review. Clin. Exp. Rheumatol..

[2] Smitten A.L., Choi H.K., Hochberg M.C., Suissa S., Simon T.A., Testa M.A., Chan K.A. (2007). The risk of herpes zoster in patients with rheumatoid arthritis in the United States and the United Kingdom. Arthritis Rheum..

[3] Chen S.Y., Suaya J.A., Li Q., Galindo C.M., Misurski D., Burstin S., Levin M.J. (2014). Incidence of herpes zoster in patients with altered immune function. Infection.

[4] Winthrop K.L., Furst D.E. (2010). Rheumatoid arthritis and herpes zoster: Risk and prevention in those treated with anti-tumour necrosis factor therapy. Ann. Rheum. Dis..

[5] Kim H., Cho S.K., Lee J., Bae S.C., Sung Y.K. (2019). Increased risk of opportunistic infection in early rheumatoid arthritis. Int. J. Rheum. Dis..

[6] Furer V., Rondaan C., Heijstek M., Van Assen S., Bijl M., Agmon-Levin N., Breedveld F.C., D’Amelio R., Dougados M., Kapetanovic M.C. (2019). Incidence and prevalence of vaccine preventable infections in adult patients with autoimmune inflammatory rheumatic diseases (AIIRD): A systemic literature review informing the 2019 update of the EULAR recommendations for vaccination in adult patients with AIIRD. Rheum. Musculoskelet. Dis. Open.

[7] Pinchinat S., Cebrián-Cuenca A.M., Bricout H., Johnson R.W. (2013). Similar herpes zoster incidence across Europe: Results from a systematic literature review. BMC Infect. Dis..

[8] Sauerbrei A. (2016). Diagnosis, antiviral therapy, and prophylaxis of varicella-zoster virus infections. Eur. J. Clin. Microbiol. Infect. Dis..

[9] John A., Canaday D.H. (2017). Herpes zoster in the older adult. Infect. Dis. Clin. N. Am..

[10] Schmader K. (2018). Herpes zoster. Ann. Intern. Med..

[11] Arvin A.M. (2008). Humoral and cellular immunity to varicella-zoster virus: An overview. J. Infect. Dis..

[12] Rondaan C., de Haan A., Horst G., Hempel J.C., van Leer C., Bos N.A., van Assen S., Bijl M., Westra J. (2014). Altered cellular and humoral immunity to varicella-zoster virus in patients with autoimmune diseases. Arthritis Rheumatol..

[13] Lang P., Aspinall R. (2021). Vaccination for quality of life: Herpes-zoster vaccines. Aging Clin. Exp. Res..

[14] Dayan R.R., Peleg R. (2017). Herpes zoster–typical and atypical presentations. Postgrad. Med..

[15] Nair P.A., Patel B.C. (2022). Herpes Zoster.

[16] Yamaguchi R., Tanaka E., Nakajima A., Inoue E., Abe M., Sugano E., Sugitani N., Saka K., Ochiai M., Higuchi Y. (2022). Risk of herpes zoster in patients with rheumatoid arthritis in the biologics era from 2011 to 2015 and its association with methotrexate, biologics, and corticosteroids. Mod. Rheumatol..

[17] Pappas D.A., Hooper M.M., Kremer J.M., Reed G., Shan Y., Wenkert D., Greenberg J.D., Curtis J.R. (2015). Herpes Zoster Reactivation in Patients with Rheumatoid Arthritis: Analysis of Disease Characteristics and Disease-Modifying Antirheumatic Drugs. Arthritis Care Res..

[18] Riley T.R., George M.D. (2021). Risk for infections with glucocorticoids and DMARDs in patients with rheumatoid arthritis. Rheum. Musculoskelet. Dis. Open.

[19] Thomas K., Sfikakis P.P., Boumpas D., Boki K., Vassilopoulos D. (2017). Study of the natural course and specific immunity after herpes zoster in patients with rheumatoid arthritis receiving biologic DMARDs. Mediterr. J. Rheumatol..

[20] Kawai K., Yawn B.P. (2017). Risk Factors for Herpes Zoster: A Systematic Review and Meta-analysis. Mayo Clin. Proc..

[21] Lecrenier N., Beukelaers P., Colindres R., Curran D., De Kesel C., De Saegher J.P., Didierlaurent A.M., Ledent E.Y., Mols J.F., Mrkvan T. (2018). Development of adjuvanted recombinant zoster vaccine and its implications for shingles prevention. Expert Rev. Vaccines.

[22] Mallick-Searle T., Snodgrass B., Brant J.M. (2016). Postherpetic neuralgia: Epidemiology, pathophysiology, and pain management pharmacology. J. Multidiscip. Healthc..

[23] Hata A., Kuniyoshi M., Ohkusa Y. (2011). Risk of herpes zoster in patients with underlying diseases: A retrospective hospital-based cohort study. Infection.

[24] Aletaha D., Neogi T., Silman A.J., Funovits J., Felson D.T., Bingham C.O., Birnbaum N.S., Burmester G.R., Bykerk V.P., Cohen M.D. (2010). 2010 rheumatoid arthritis classification criteria: An American College of Rheumatology/European League Against Rheumatism collaborative initiative. Ann. Rheum. Dis..

[25] Johnson R.W., Alvarez-Pasquin M.J., Bijl M., Franco E., Gaillat J., Clara J.G., Labetoulle M., Michel J.P., Naldi L., Sanmarti L.S. (2015). Herpes zoster epidemiology, management, and disease and economic burden in Europe: A multidisciplinary perspective. Ther. Adv. Vaccines.

[26] McDonald J.R., Zeringue A.L., Caplan L., Ranganathan P., Xian H., Burroughs T.E., Fraser V.J., Cunningham F., Eisen S.A. (2009). Herpes zoster risk factors in a national cohort of veterans with rheumatoid arthritis. Clin. Infect. Dis..

[27] Wolfe F., Michaud K., Chakravarty E.F. (2006). Rates and predictors of herpes zoster in patients with rheumatoid arthritis and non-inflammatory musculoskeletal disorders. Rheumatology.

[28] Harada S., Sakai R., Hirano F., Miyasaka N., Harigai M. (2017). Association Between Medications and Herpes Zoster in Japanese Patients with Rheumatoid Arthritis: A 5-year Prospective Cohort Study. J. Rheumatol..

[29] Youssef J., Novosad S.A., Winthrop K.L. (2016). Infection Risk and Safety of Corticosteroid Use. Rheum. Dis. Clin. N. Am..

[30] Strangfeld A., Listing J., Herzer P., Liebhaber A., Rockwitz K., Richter C., Zink A. (2009). Risk of herpes zoster in patients with rheumatoid arthritis treated with anti-TNF-alpha agents. J. Am. Med. Assoc..

[31] Pawar A., Desai R.J., Solomon D.H., Ortiz A.J., Gale S., Bao M., Sarsour K., Schneeweiss S., Kim S.C. (2019). Risk of serious infections in tocilizumab versus other biologic drugs in patients with rheumatoid arthritis: A multidatabase cohort study. Ann. Rheum. Dis..

[32] Yun H., Xie F., Delzell E., Chen L., Levitan E.B., Lewis J.D., Saag K.G., Beukelman T., Winthrop K., Baddley J.W. (2015). Risks of Herpes Zoster in Patients with Rheumatoid Arthritis According to Biologic Disease-Modifying Therapy. Arthritis Care Res..

[33] Trana C.T., Ducancellea A., Massonb C., Lunel-Fabiani F. (2017). Herpes zoster: Risk and prevention during immunomodulating therapy. Jt. Bone Spine.

[34] Sunzini F., McInnes I., Siebert S. (2020). JAK inhibitors and infections risk: Focus on herpes zoster. Ther. Adv. Musculoskelet. Dis..

[35] Sunzini F., McInnes I., Siebert S. (2017). Patient-reported outcomes from a randomised phase III study of baricitinib in patients with rheumatoid arthritis and an inadequate response to biological agents (RA-BEACON). Ann. Rheum. Dis..

[36] Fleischmann R., Mysler E., Hall S., Kivitz A.J., Moots R.J., Luo Z., DeMasi R., Soma K., Zhang R., Takiya L. (2017). Efficacy and safety of tofacitinib monotherapy, tofacitinib with methotrexate, and adalimumab with methotrexate in patients with rheumatoid arthritis (ORAL Strategy): A Phase IIIb/IV, double-blind, head-to-head, randomised controlled trial. Lancet.

[37] Burmester G.R., Kremer J.M., Van den Bosch F., Kivitz A., Bessette L., Li Y., Zhou Y., Othman A.A., Pangan A.L., Camp H.S. (2018). Safety and efficacy of upadacitinib in patients with rheumatoid arthritis and inadequate response to conventional synthetic disease-modifying anti-rheumatic drugs (SELECT-NEXT): A randomised, double-blind, placebo-controlled phase 3 trial. Lancet.

[38] Fleischmann R., Pangan A.L., Song I.H., Mysler E., Bessette L., Peterfy C., Durez P., Ostor A.J., Li Y., Zhou Y. (2019). Upadacitinib versus placebo or adalimumab in patients with rheumatoid arthritis and an inadequate response to methotrexate: Results of a phase 3, double-blind, randomized controlled trial. Arthritis Rheumatol..

[39] Chen Y.J., Chen Y.M., Huang W.N., Chen H.H., Liao T.L., Chen J.P., Hsieh T.Y., Chen Y.H., Chen D.Y. (2020). Herpes Zoster in rheumatoid arthritis patients receiving tofacitinib, a single center experience from Taiwan. Medicine.

[40] Bechman K., Subesinghe S., Norton S., Atzeni F., Galli M., Cope A.P., Winthrop K.L., Galloway J.B. (2019). A systematic review and meta-analysis of infection risk with small molecule JAK inhibitors in rheumatoid arthritis. Rheumatology.

[41] Sullivan N.L., Eberhardt C.S., Wieland A., Vora K.A., Pulendran B., Ahmed R. (2019). Understanding the immunology of the Zostavax shingles vaccine. Curr. Opin. Immunol..

[42] Shah R.A., Limmer A.L., Nwannunu C.E., Patel R.R., Mui U.N., Tyring S.K. (2019). Shingrix for Herpes Zoster: A Review. Ski. Ther. Lett..

[43] Furer V., Rondaan C., Heijstek M.W., Agmon-Levin N., Van Assen S., Bijl M., Breedveld F.C., D’amelio R., Dougados M., Kapetanovic M.C. (2020). 2019 update of EULAR recommendations for vaccination in adult patients with autoimmune inflammatory rheumatic diseases. Ann. Rheum. Dis..

