# Supplementary material for: Increased Risk of Herpes Zoster in Rheumatoid Arthritis Not Only Due to JAK Inhibitors—Study of 392 Patients from Single University Center
Source: J Clin Med. 2024 May 26;13(11):3121. doi: 10.3390/jcm13113121 (PMC11172981; doi:10.3390/jcm13113121)
Supplement: Supplementary file 1 [file jcm-13-03121-s001.zip › jcm-2937251-supplementary.pdf]

Supplementary Table S1. Herpes zoster infection characteristics and therapy for rheumatoid arthritis when infection appeared.

**Age at the moment of HZ,mean±SD (years)**	64.7±11.8
**Time of evolution of RA, mean±SD (months)**	158.4±115.7
Location, n(%)	
Trunk	18 (60)
Head and neck	4 (13)
Extremities	2 (7)
Disseminated	1 (3)
Unknown	5 (17)
**Antiviral treatment, n (%)**	23 (77)
Brivudine	7 (30)
Famciclovir	7 (30)
Aciclovir	6 (126)
Valaciclovir	3 (13)
Sequelae	
Postherpetic neuralgia	7 (87)
Temporary visual alteration	1 (13)
Current treatment when zoster infection occurred	
Prednisone, n (%)	19 (63)
Prednisonedose, mg/day	7.5 (5-10)
DMARDs, n (%)	15 (50)
Metothrexate	10 (67)
Leflunomide	2 (13)
Metothrexate + leflunomide	1 (7)
Sulfasalazine	1 (7)
Biologicaltherapy, n (%)	15 (50)
Etanercept	4 (27)
Abatacept	3 (20)
Tocilizumab	3 (20)
Golimumab	2 (13)

Adalimumab	1 (7)
Certolizumab	1 (7)
Sarilumab	1 (7)
JAK inhibitors, n (%)	3 (10)
Tofacitinib	2 (13)
Upadacitinib	1 (33)

Abbreviations (in alphabetical order): DMARDs: disease-modifying anti-rheumatic drugs; HZ: herpes zoster; JAK: Janus kinase; N (n): number; RA: rheumatoid arthritis: SD: standard deviation.
